# Comparative Study of Lumbar Disc Prolapse Surgery With or Without Coagulation: A Report of 2,180 Cases

**DOI:** 10.7759/cureus.92903

**Published:** 2025-09-22

**Authors:** Md Moshiur Rahman, S.I.M. Khairun Nabi Khan, Robert Ahmed Khan

**Affiliations:** 1 Neurosurgery Department, Holy Family Red Crescent Medical College Hospital, Dhaka, BGD; 2 Neurosurgery Department, Bangabandhu Sheikh Mujib Medical University (BSMMU), Dhaka, BGD

**Keywords:** coagulation, lumbar disc prolapse, macnab’s criteria, microlumbar discectomy, minimally invasive spine surgery, promis score, surgical outcomes

## Abstract

Lumbar disc prolapse surgery is among the most common neurosurgical procedures performed worldwide. To our knowledge, this is the first report of the largest series comparing prolapsed lumbar disc surgery with and without coagulation. Previous studies have shown that coagulation during surgery may be associated with higher morbidity, particularly in terms of infection rates. This study aims to investigate the necessity of coagulation during lumbar microdiscectomy, specifically whether coagulation is required to control bleeding in the disc space, surrounding ligaments, or other tissues during the procedure.

A total of 2,180 cases were retrospectively analyzed from 2010 to 2020, with a minimum follow-up of two years. The study compared the effect of coagulation on outcomes for lumbar microdiscectomy, specifically evaluating quality of life through the PROMIS (Patient-Reported Outcomes Measurement Information System) score. PROMIS was used to assess both physical and mental health outcomes before and after surgery. The data were collected from a single center.

Patients were divided into two groups: Group 1 (coagulation) and Group 2 (no coagulation). The PROMIS score was obtained preoperatively and postoperatively using the online “ortho-toolkit” questionnaires, and the average scores for both groups were calculated to assess their overall health status. The average preoperative physical health score (PHS T-score) was 32.4 for Group 1 and 31.3 for Group 2. Postoperatively, the scores were 54.1 for Group 1 and 56.2 for Group 2. Similarly, the average preoperative mental health score (MHS T-score) was 41.1 for Group 1 and 39.7 for Group 2, with postoperative scores of 48.3 for Group 1 and 52.8 for Group 2.

Lumbar disc prolapse surgery without coagulation is safe and effective. There is no difference in outcome using coagulation or not in lumbar disc prolapse surgery, and its use is not obligatory.

## Introduction

Lumbar disc herniation (LDH) occurs when the intervertebral disc in the lower spine protrudes or ruptures into the spinal canal. While many cases respond to conservative treatment, a subset of patients with persistent pain, progressive neurological deficits, or functional disability ultimately require surgery.

The incidence of lumbar disc prolapse is rising, partly due to increasing life expectancy and occupational stress [1]. Numerous studies have examined surgical versus non-surgical management of herniated discs, but many were limited by small sample sizes, differences in baseline patient characteristics, and lack of standardized outcome measures [2-4]. Minimally invasive spine procedures generally offer shorter hospital stays, quicker return to work, and favorable safety profiles, particularly for elderly patients with comorbidities [5-7]. Technological advances in optics, surgical tools, and techniques have further improved the feasibility of endoscopic and microscopic approaches [8,9].

In this study, we evaluated the necessity of coagulation in standard microlumbar discectomy. The procedure was performed using a tubular retractor, and diathermy coagulation was deliberately avoided. Instead, alternative hemostatic methods were applied, which are considered effective and less invasive, reducing the risk of thermal injury. To date, no large randomized trial has directly compared the outcomes of coagulation versus non-coagulation in lumbar disc prolapse surgery. Therefore, we present a large cohort analysis with a two-year follow-up, assessing outcomes using the Patient-Reported Outcomes Measurement Information System (PROMIS) scoring system to evaluate the impact of coagulation on patient recovery and quality of life. In this comparative study, one group had all forms of coagulation (monopolar and bipolar), while the other group had no coagulation applied from skin to skin.

## Materials and methods

A total of 2,180 cases were retrospectively reviewed from 2010 to 2020. All patients had a minimum follow-up period of two years. The study compared the effect of using or avoiding coagulation during surgery for a prolapsed lumbar intervertebral disc (PLID) on postoperative quality of life. The PROMIS score was used to assess clinical outcomes before and after surgery.

Inclusion criteria

Patients were included if they had symptomatic lumbar disc prolapse and had failed conservative treatment, including analgesics, rest, and physiotherapy, for a period of six to eight weeks.

Exclusion criteria

Patients were excluded if they had cauda equina syndrome, multilevel lumbar spinal stenosis, a coagulation defect, or a history of previous lumbar surgery.

Intervention

Operative Procedure

In the intervention group, standard microlumbar discectomy for lumbar disc prolapse was performed using a tubular retractor. Throughout the procedure, diathermy coagulation was not used to control bleeding from the disc space, ligaments, or surrounding tissues. Alternative hemostatic methods, such as bone wax for bony bleeding and cottonoids for epidural bleeding, were used. A closed suction drain was placed to drain epidural bleeding and kept in situ for not more than 48 hours.

Control or Comparator

The comparator group included patients with symptomatic lumbar disc prolapse who underwent surgery where diathermy coagulation was routinely used for hemostasis, including monopolar and bipolar coagulation to control bleeding during surgery.

Statistical analysis

All statistical analyses were performed using SPSS 27 (IBM Corp., Armonk, NY, US). Descriptive statistics were calculated for continuous variables such as age, symptom duration, operative time, hospital stay, and return to work, and these were expressed as mean ± standard deviation (SD). Categorical variables such as sex, disc level involvement, complications, recurrence, and outcome according to Macnab’s criteria were presented as frequencies and percentages (N (%)). Comparisons between groups (coagulation vs. no coagulation) were carried out using the independent samples t-test for continuous variables. Preoperative and postoperative PROMIS physical health score (PHS) and mental health score (MHS) within each group were analyzed using the paired t-test. A p-value of <0.05 was considered statistically significant for all comparisons of demographic data, baseline clinical features, and perioperative parameters. The primary outcome was the change in PROMIS Global-10 Physical Health and Mental Health T-scores before and after surgery in each group. Secondary outcomes included the complication rate, recurrence rate, and functional outcome according to Macnab’s criteria.

## Results

The demographic characteristics of the patients are presented in Table 1. The mean age of the study population was 47.8 ± 3.7 years (p > 0.05). Men accounted for 54.8% (n = 1,194) and women 45.2% (n = 986) of the cohort (p > 0.05). The most frequently affected disc level was L4-L5 (45%), followed by L5-S1 (34%), while the least affected was L1-L2 (3%) (p > 0.05). The mean operative time was 31.7 ± 6.1 minutes, the mean hospital stay was 1.4 ± 2.3 days, and the mean time to return to work was 14.7 ± 72.4 days. Complications occurred in 8.9% of cases and recurrences in 6.2% of cases (p > 0.05).

**Table 1:** Demographic data of all patients Values are presented as mean ± SD for continuous variables and N (%) for categorical variables. A p-value < 0.05 was considered statistically significant. SD: standard deviation

Characteristics	Values	p-value
Mean age (years)	47.8 ± 3.7	>0.05
Sex: male	1,194 (54.8%)	>0.05
Sex: female	986 (45.2%)	>0.05
Level of disc: L1–L2	66 (3.0%)	>0.05
Level of disc: L2–L3	66 (3.0%)	>0.05
Level of disc: L3–L4	330 (15.0%)	>0.05
Level of disc: L4–L5	990 (45.0%)	>0.05
Level of disc: L5–S1	748 (34.0%)	>0.05
Mean symptom duration (0–3 months)	58.4 ± 45.7	>0.05
Mean symptom duration (3–6 months)	55.1 ± 5.3	>0.05
Mean symptom duration (6–9 months)	56.9 ± 4.1	>0.05
Mean operative time (minutes)	31.7 ± 6.1	>0.05
Mean hospital stay (days)	1.4 ± 2.3	>0.05
Mean time to return to work (days)	14.7 ± 72.4	>0.05
Complications	196 (8.9%)	>0.05
Recurrences	136 (6.2%)	>0.05

The distribution of symptom presentation is summarized in Table 2. More than half of the patients presented with low back pain radiating to the leg (53.3%). Sensory impairments were reported by 38.3% of patients, while motor deficit was observed in 15.8% of cases.

**Table 2:** Mode of symptom presentation of patients Percentages are calculated from the total study population (N = 2,180).

Presentation	Number of patients	Percentage (%)
Low back pain with radiation to the leg	1,163	53.3
Low back pain with sensory impairments	836	38.3
Low back pain with motor deficit	345	15.8

Clinical outcomes based on Macnab’s criteria are shown in Table 3. An excellent outcome was achieved in 51.2% of patients, while 41.4% had a good outcome. Fair and poor outcomes were observed in 4.6% and 2.8% of patients, respectively.

**Table 3:** Outcome of patients according to Macnab’s criteria Outcomes were assessed according to Macnab’s criteria. Values are presented as N (%).

Macnab’s criteria	Number of patients	Percentage (%)
Excellent	1,116	51.2
Good	902	41.4
Fair	101	4.6
Poor	61	2.8
Total	2,180	100.0

Primary outcome(s)

**Figure 1:**
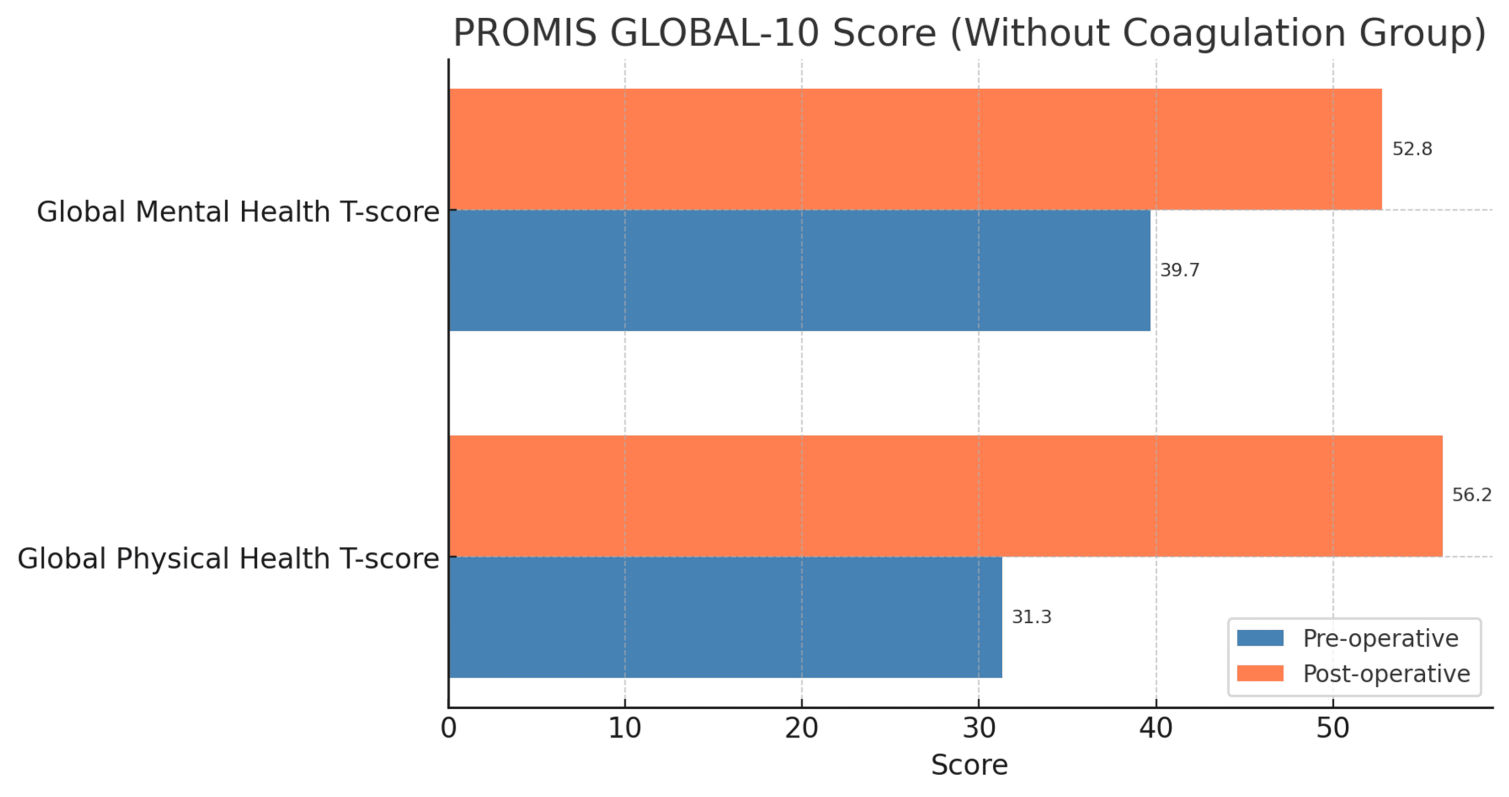
Comparison of health status according to the PROMIS GLOBAL-10 Score (without coagulation group) PROMIS: Patient-Reported Outcomes Measurement Information System

**Figure 2:**
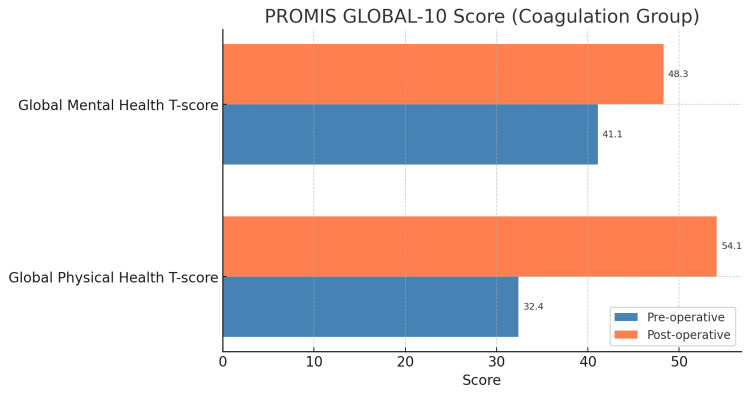
Comparison of health status according to the PROMIS GLOBAL-10 Score (coagulation group) PROMIS: Patient-Reported Outcomes Measurement Information System

Patients were divided into two groups (Group 1: coagulation group and Group 2: without coagulation group). We got the PROMIS score pre- and postoperatively using online “ortho-toolkit” questionnaires and calculated patients’ average scores in both groups. We used it to know the overall health status of both groups. The average preoperative values of the PHS T-score were 32.4 (Group 1) and 31.3 (Group 2), and postoperatively, the values were 54.1 (Group 1) and 56.2 (Group 2) (Figures 1, 2).

Key secondary outcomes

Similarly, the average preoperative values of the MHS T-score were 41.1 (Group 1) and 39.7 (Group 2), and the postoperative values were 48.3 (Group 1) and 52.8 (Group 2), respectively (Figures 1, 2).

## Discussion

Coagulation techniques during lumbar disc prolapse surgery play a crucial role in ensuring optimal outcomes. While patient selection is essential for successful surgery, the impact of coagulation on postoperative recovery and quality of life has been less explored. Studies have suggested that coagulation, particularly the use of monopolar or bipolar methods, can affect intraoperative bleeding control and postoperative healing. However, there is limited literature directly comparing the outcomes of coagulation versus non-coagulation in PLID surgeries. This study aims to address this gap by evaluating the effect of coagulation on patient recovery using the PROMIS scoring system to assess both physical and mental health outcomes [10,11]. In actuality, pre-operative neurological conditions and imaging studies can be used to evaluate the majority of surgical failure cases. The surgeon must always be aware of his technical limits in light of the patient's clinical and radiological circumstances in order to choose the right patient. Intent-to-treat analyses showed no significant differences between the randomized groups on the important end-measures, and patients in both groups showed considerable improvement over time in that experiment. Depending on the skill or expertise of the surgeon, surgical indications may change [12,13]. By using imaging tests like an X-ray, CT scan, or MRI, surgeons can determine the type of disc herniation as well as the viability of the minimally invasive procedure. Nerve root deviation and herniation type can be assessed with preoperative MRIs. It is important to evaluate the degree of migration, the degree of disc herniation, the degree of adhesion, the risk of a dural rupture, the softness of the herniated disc, and any concurrent spinal stenosis. L4/5 (45%) was the most affected disc level in our study, while L1-L2 (p > 0.05) was the least affected disc level. Preoperative strategies and surgical techniques should be customized for each patient's unique ruptured disc migration pattern [14]. Successful lumbar disc prolapse is influenced by the specifics of the lesion and is impacted by appropriate surgical planning, including method selection. Determining the proper spinal level for surgery and avoiding surgical and procedural errors depend on the preoperative detection of a disc prolapse. Using Macnab's criteria, we assessed the health outcomes of the patients in our study. In our study, 51.2% of patients had great results, 41.4% had acceptable results, 4.6% had medium results, and 2.8% had bad results. Approximately 94.09% of patients reported good and satisfactory outcomes for lower limb and low back pain, according to a study based on modified Macnab’s ratings [15]. Additionally, we used the PROMIS score to determine each group's general health status. Groups 1 and 2 had average preoperative PHS T-scores of 32.4 and 31.3, respectively, and postoperative values of 54.1 and 56.2, respectively. Likewise, the mean preoperative MHS T-score was 41.1 for Group 1 and 39.7 for Group 2, and postoperative values of 48.3 for Group 1 and 52.8 for Group 2, respectively. After surgical repair, recurrent disc herniation remains a concern; 8.9% of patients experienced complications, while 6.2% experienced recurrences. According to a meta-analysis of 63 trials that used percutaneous endoscopic lumbar discectomy (PELD) for LDH, the overall pooled prevalence was 3.6%, while the reported rates of recurrence varied from 0% to 12.5% [16]. After a microdiscectomy, the chance of recurrence ranges from 5% to over 20% [14]. Due to postoperative epidural scarring, increasing degeneration with stenosis, arachnoiditis, segmental instability, or further tissue damage, the success rate of revisional lumbar disc prolapse surgery may be lower than that of the original procedure [17]. Tissue damage and its related effects can be reduced with minimally invasive techniques [18,19]. Innovative endoscopic techniques with similar clinical results and fewer complications than open and microscopic surgery have been made possible by endoscopic technology [19,20].

Limitations

Our study's primary limitation is its retrospective design, which may introduce certain biases, including selection bias and confounding factors. Additionally, some patients were lost to follow-up two years post-surgery, which may have impacted the completeness and accuracy of the data. While we made efforts to control for these factors, the results should be interpreted with caution. Furthermore, the absence of a randomized controlled design limits the ability to establish definitive causality between coagulation methods and surgical outcomes.

Future directions

To corroborate our findings and address the limitations of this study, future research should focus on prospective, randomized controlled trials. These studies would help to eliminate biases associated with retrospective designs and offer more robust evidence regarding the impact of coagulation on outcomes in lumbar disc prolapse surgery. Additionally, longer follow-up periods and larger sample sizes would provide more comprehensive insights into the long-term effects of coagulation on patient recovery and quality of life.

## Conclusions

In this study, we found that performing lumbar disc prolapse surgery without coagulation yields results comparable to those achieved with coagulation. Our analysis of 2,180 cases, with a minimum follow-up of two years, showed no statistically significant differences between the two groups in terms of complication rate, recurrence, or patient-reported quality of life outcomes. Both groups demonstrated significant improvements in PHS and MHS post-surgery, as assessed by the PROMIS scoring system.

These findings suggest that the routine use of coagulation is not mandatory in PLID surgery and can be safely omitted without compromising the surgical outcomes. Eliminating coagulation may also reduce the potential risks associated with thermal tissue damage and infection due to diathermy. Therefore, the decision to use coagulation should be based on the surgeon’s judgment, taking into account the patient’s condition and the intraoperative context, rather than being a required step. Further randomized, prospective, multicenter studies are necessary to confirm these findings and provide more robust evidence that could help standardize surgical protocols in the management of lumbar disc prolapse.
